# Prolonged Time to Adjuvant Chemotherapy Initiation Was Associated with Worse Disease Outcome in Triple Negative Breast Cancer Patients

**DOI:** 10.1038/s41598-020-64005-4

**Published:** 2020-04-27

**Authors:** Lifen Cai, Yiwei Tong, Xiaoping Zhu, Kunwei Shen, Juanying Zhu, Xiaosong Chen

**Affiliations:** 1 Department of Breast, Jiaxing University Affiliated Women and Children Hospital, Jiaxing, China; 2 Comprehensive Breast Health Center, Ruijin Hospital, Shanghai Jiao Tong University School of Medicine, Shanghai, China

**Keywords:** Breast cancer, Chemotherapy

## Abstract

The optimal time to adjuvant chemotherapy (TTC) for breast cancer (BC) patients remains uncertain. Herein, we aim to evaluate the association between TTC and prognosis among different subtypes in modern era of adjuvant chemotherapy. BC patients receiving operation and adjuvant chemotherapy between January 2009 and December 2015 were included. Enrolled patients were categorized into TTC ≤4 weeks and >4 weeks groups. Relapse-free survival (RFS) and overall survival (OS) were compared according to TTC and analyzed among different BC molecular subtypes. A total of 2611 patients were included. Elder age (*P* = 0.005), more comorbidities (*P* <0.001), breast-conserving surgery (*P* = 0.001), non-invasive ductal carcinoma (*P* = 0.012), and HER2-positivity (*P <*0.001) were associated with prolonged TTC. Among whole BC population, no significant difference was observed between two TTC groups in terms of RFS (*P* = 0.225) or OS (*P* = 0.355). However, for triple negative (TNBC) patients, TTC >4 weeks was independently related with worse RFS (5-year RFS 81.9% *vs* 89.3%; HR, 1.89; 95% CI, 1.09 to 3.27; *P* = 0.024) and OS (5-year OS 84.0% *vs* 94.0%; HR, 2.49; 95% CI, 1.30 to 4.76; *P* = 0.006) compared with those TTC ≤4 weeks. Prolonged TTC >4 weeks after BC surgery was not associated with worse survival outcomes in the whole BC patients. However, TTC >4 weeks may increase risk of relapse or death in TNBC patients, which deserves further clinical evaluation.

## Introduction

Breast cancer (BC) is the most common malignancy in women worldwide^1^. Chemotherapy is an essential part of systemic treatment in early BC patients. Adjuvant chemotherapy has been demonstrated to decrease the risk of disease recurrence by 30% to 50%, which corresponded to an absolute overall survival (OS) benefit of 10%^2,3^. As a heterogeneous disease, BC is classified into different molecular subtypes according to its estrogen receptor (ER), progesterone receptor (PR), human epidermal growth factor receptor 2 (HER2), and Ki67 status^4^. Triple negative breast cancer (TNBC) is one of the subtypes which is defined by the absence of ER, PR and HER2 overexpression. Representing 15% of BC, TNBC is featured by its aggressive biology with a higher risk of local and distant relapses^5^. Currently, chemotherapy is the only routinely used effective systemic treatment for TNBC patients.

The timing of adjuvant chemotherapy initiation after breast cancer surgery may influence adjuvant treatment efficacy. Preclinical studies in animal models suggested that adjuvant chemotherapy should be applied as early as possible following the removal of tumor so as to gain the greatest benefit^6,7^. However, the optimal interval from definitive surgery to the initiation of adjuvant chemotherapy (time to adjuvant chemotherapy, TTC) remains uncertain^8^. TTC intervals reported in previous studies ranged from 2 to 12 weeks^8–12^. A meta-analysis showed that each 4-week delay in TTC would lead to a 15% decrease in OS and a 16% decrease in disease-free survival (DFS), respectively^13^. Moreover, the influence of chemotherapy delay on survival was subtype-dependent^9,10^, which was particularly relevant in TNBC patients^11,12^. Nevertheless, there were conflicting results coming from other studies^9–12,14,15^. Jara Sánchez *et al*. found no difference in terms of OS or DFS when comparing patients with TTC <3 weeks, 3–6 weeks, 6–9 weeks and ≥9 weeks^16^. They even demonstrated an increased DFS with increased TTC in ER-negative patients. The impact of TTC on BC patient survival remained controversial.

As a result, we aim to evaluate the general TTC in Chinese population receiving modern era of adjuvant chemotherapy, to identify potential impact factors for prolonged TTC, and then to establish the association between TTC and prognosis among BC patients with different molecular subtypes.

## Patients and methods

### Patient population

Invasive BC patients who received surgery from January 2009 and December 2015 in the Department of Breast, Jiaxing University Affiliated Women and Children Hospital or Comprehensive Breast Health Center, Ruijin Hospital, Shanghai Jiao Tong University School of Medicine were retrospectively included if they met the following criteria: (1) stage I to III disease; (2) female gender; (3) with available clinico-pathological, immunohistochemistry (IHC) or fluorescence *in situ* hybridization (FISH) results to construct molecular subtypes; (4) receiving standard adjuvant chemotherapy; (5) with complete follow-up information. Those who received preoperative systematic treatment or diagnosed with *de novo* stage IV disease were excluded. This study was approved by the independent Ethical Committees of Jiaxing University Affiliated Women and Children Hospital, and Ruijin Hospital, Shanghai Jiao Tong University School of Medicine.

Data were obtained from Shanghai Jiao Tong University Breast Cancer Database (SJTU-BCDB) on age at diagnosis, comorbidities, breast surgery type, histologic type, histologic grade, pathological tumor size, pathological nodal status, lymphovascular invasion (LVI), tumor IHC evaluation including ER, PR, HER2, and Ki67 status. The cutoff value for ER and PR positivity was no less than 1% positive invasive cell with nuclear staining^17^. HER2 status was determined according to the ASCO/CAP (American Society of Clinical Oncology/College of American Pathologists) testing guidelines at the time of surgery^18^. BC molecular subtype was defined with regards to the 2013 St. Gallen consensus^4^ as Luminal A (ER-positive, HER2-negative, Ki67 <14% and PR ≥20%), Luminal B/HER2-negative (hormone receptor [HR] positive, HER2-negative, Ki67 ≥14% or PR <20%), Luminal B/HER2-positive (HR-positive and HER2-positive), HER2-positive (HR-negative and HER2-positive), and TNBC (HR-negative and HER2-negative).

### Treatment decision

A multidisciplinary team (MDT) meeting, which included surgical oncologists, medical oncologists, radiation oncologists, pathologists, BC specialized nurses, and other related specialists was held to decide adjuvant treatment regimens based on patient’s clinico-pathological characteristics. Frequently proposed chemotherapy regimens included A (anthracyclines-containing), T (taxanes-containing), A + T (anthracyclines and taxanes-containing), and others (such as platinum-based regimen, capecitabine, *etc*.). Patient compliance, actual chemotherapy usage, and regimen were confirmed by BC specialized nurses during follow-up.

### Follow-up

Patient follow-up was conducted by BC specialized nurses in each center. Relapse-free survival (RFS) was measured from the date of surgery to the date of first documented local or distant recurrence or death due to any cause. OS was measured from the date of surgery to the date of death of any cause. The diagnosis of recurrence was generally based on radiographic images and/or pathologic biopsies when accessible. Last follow-up was completed in June 2019.

### Statistical analysis

TTC was defined from the definitive surgery to the initiation of adjuvant chemotherapy. Patients were then categorized according to TTC length into two groups: ≤4 weeks and >4 weeks. Patient characteristics were compared according to TTC categories by using Chi-square test or Fisher’s exact test. Charlson Comorbidity Index (CCI) was applied to evaluate patient comorbidity. Odds ratio (OR) with 95% confidence interval (CI) were calculated from multivariate logistic regression analysis to identify impact factors for TTC. Kaplan-Meier curve was adopted to estimate the 5-year RFS and 5-year OS according to TTC and patient characteristics. Subgroup analysis was conducted using stratified Mantel-Haenszel test to estimate hazard ratio (HR) with 95% CI. Cox proportional hazards regression models was applied to determine the association between TTC and survival outcomes after adjustment for potential confounders. Two-sided *P* values < 0.05 were considered statistically significant. Statistical analyses were carried out by using the IBM SPSS statistics software version 25.0 (SPSS, Inc., IL, USA) and GraphPad Prism version 7.0 (GraphPad Software, CA, USA).

## Results

### Patient baseline characteristics

In all, 2611 BC patients were included. Patient characteristics by TTC groups were listed in Table 1. The median age of the study population was 52 (interquartile range [IQR] 45–60) years. CCI was 0, 1, and ≥2 in 1869, 537, and 205 patients, respectively. There were 76.2% patients who received mastectomy with (2.2%) or without (74.0%) immediate breast reconstruction, while 23.8% patients underwent breast-conserving surgery (BCS). A total of 2386 patients (91.4%) were diagnosed with invasive ductal carcinoma (IDC). There were 48.8% and 42.8% patients with grade I-II or III tumors. Tumor size was pT1, pT2, and pT3–4 in 55.4%, 42.4%, and 2.3% patients, respectively. Axillary lymph node involvement was confirmed in 42.5% patients. Only 7.8% patients had LVI. The positivity rates of ER, PR, and HER2 were 66.3%, 52.1%, and 26.0%. There were 13.4%, 40.9%, 12.1%, 19.7%, and 13.9% patients classified with Luminal A, Luminal B/HER2-negative, Luminal B/HER2-positive, TNBC, and HER2-positive subtype, respectively. Baseline features of the patients treated in the Department of Breast, Jiaxing University Affiliated Women and Children Hospital and those treated in the Comprehensive Breast Health Center, Ruijin Hospital, Shanghai Jiao Tong University School of Medicine were compared and presented in Supplementary Table S1.

**Table 1:** Patient baseline characteristics by TTC.

Characteristics	All Patients (n = 2611)	TTC (weeks)		*P*
		≤4 (n = 1900)	>4 (n = 711)	
	No. (%)	No. (%)	No. (%)	
**Age at diagnosis, years**				
Median age (IQR)	52 (45–60)	51 (45–59)	55 (47–62)	
<50	1065 (40.8)	823 (43.3)	242 (34.0)	<0.001
≥50	1546 (59.2)	1077 (56.7)	469 (66.0)	
**Comorbidity**^**†**^				<0.001
0	1869 (71.6)	1411 (74.3)	458 (64.4)	
1	537 (20.6)	365 (19.2)	172 (24.2)	
≥2	205 (7.9)	124 (6.5)	81 (11.4)	
**Breast surgery**				0.008
Mastectomy*	1989 (76.2)	1473 (77.5)	516 (72.6)	
BCS	622 (23.8)	427 (22.5)	195 (27.4)	
**Histologic type**				0.046
IDC	2386 (91.4)	1749 (92.1)	637 (89.6)	
Non-IDC	225 (8.6)	151 (7.9)	74 (10.4)	
**Histologic grade**				0.102
I-II	1275 (48.8)	951 (50.1)	324 (45.6)	
III	1118 (42.8)	798 (42.0)	320 (45.0)	
Unknown	218 (8.3)	151 (7.9)	67 (9.4)	
**Pathological tumor size**				0.338
T1	1446 (55.4)	1066 (56.1)	380 (53.4)	
T2	1106 (42.4)	789 (41.5)	317 (44.6)	
T3–4	59 (2.3)	45 (2.4)	14 (2.0)	
**Pathological node status**				0.374
Negative	1502 (57.5)	1083 (57.0)	419 (58.9)	
Positive	1109 (42.5)	817 (43.0)	292 (41.1)	
**LVI**				0.012
Negative	2408 (92.2)	1737 (91.4)	671 (94.4)	
Positive	203 (7.8)	163 (8.6)	40 (5.6)	
**ER status**				0.128
Negative	879 (33.7)	656 (34.5)	223 (31.4)	
Positive	1732 (66.3)	1244 (65.5)	488 (68.6)	
**PR status**				0.818
Negative	1250 (47.9)	907 (47.7)	343 (48.2)	
Positive	1361 (52.1)	993 (52.3)	368 (51.8)	
**HER2 status**				<0.001
Negative	1932 (74.0)	1449 (76.3)	483 (67.9)	
Positive	679 (26.0)	451 (23.7)	228 (32.1)	
**Ki67, %**				0.153
<14	763 (29.2)	570 (30.0)	193 (27.1)	
≥14	1848 (70.8)	1330 (70.0)	518 (72.9)	
**Molecular subtype**				<0.001
Luminal A	349 (13.4)	273 (14.4)	76 (10.7)	
Luminal B/HER2-negative	1068 (40.9)	762 (40.1)	306 (43.0)	
Luminal B/HER2-positive	317 (12.1)	210 (11.1)	107 (15.0)	
TNBC	515 (19.7)	414 (21.8)	101 (14.2)	
HER2-positive	362 (13.9)	241 (12.7)	121 (17.0)	

Abbreviations: TTC, time to adjuvant chemotherapy; IQR, interquartile range; BCS, breast-conserving surgery; IDC, invasive ductal carcinoma; LVI, lymphovascular invasion; ER, estrogen receptor; PR, progesterone receptor; HER2, human epidermal growth factor receptor 2; TNBC, triple negative breast cancer.

^†^Comorbidity score was calculated according to Charlson Comorbidity Index.

*57 (2.2%) out of 1989 patients received mastectomy + immediate breast reconstruction.

Adjuvant treatment recommendations were listed in Supplementary Table S2. The most frequently used regimens were EC-T (epirubicin 90 mg/m^2^ and cyclophosphamide 600 mg/m^2^ every 21 days followed by docetaxcel 80–100 mg/m^2^ every 21 days or weekly paclitaxel 80 mg/m^2^), TC (docetaxel 75 mg/m^2^ plus cyclophosphamide 600 mg/m^2^ every 21 days), and EC (epirubicin 90 mg/m^2^ and cyclophosphamide 600 mg/m^2^ every 21 days) in the whole population. Chemotherapy regimens were differently distributed among different molecular subtypes (*P* < 0.001, Supplementary Figure S1). Patients receiving EC-T regimen tended to start adjuvant chemotherapy sooner after surgery (*P* < 0.001, Supplementary Table S2). However, chemotherapy regimen was not an independent impact factor related to TTC in the multivariate analysis (*P* = 0.187). Patients treated with targeted therapy was more likely to start chemotherapy later (*P* < 0.001, Supplementary Table S2). Radiation therapy and endocrine therapy recommendation were comparable between two TTC groups.

### Factors associated with TTC

**Figure 1:**
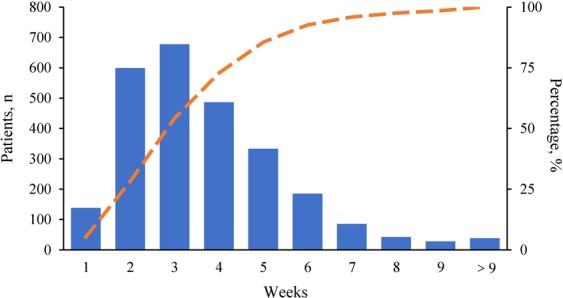
Distribution of TTC in the study population. The histogram bars refer to number of patients, as indicated by left y-axis. The orange dashed line indicates cumulative percentage of patients who received chemotherapy, as indicated by right y-axis. Abbreviations: TTC, time to adjuvant chemotherapy.

The distribution of TTC in the study population was demonstrated in Fig. 1. The median TTC was 20 (IQR 13–29) days. There were 72.8% patients receiving chemotherapy within 4 weeks after surgery (TTC ≤4 weeks). The other 27.2% patients received chemotherapy more than 4 weeks after surgery (TTC> 4 weeks). Univariate analysis found that age, comorbidities, breast surgery type, histologic type, LVI, HER2-status, and molecular subtype were significantly different between two TTC groups (all *P* < 0.05, Table 1).

Multivariate analysis demonstrated that age, comorbidities, breast surgery type, histologic type, and molecular subtype were independently associated with TTC >4 weeks (Table 2). Those who experienced prolonged TTC were more likely to be ≥50 years (*P* = 0.005), and with comorbidities (CCI = 1: *P* = 0.004; CCI ≥2: *P <*0.001). BCS procedure (*P* = 0.001) and non-IDC tumor (*P* = 0.012) also led to TTC delay. In addition, patients with Luminal B/HER2-positive subtype (*P* = 0.002) and HER2-positive subtype (*P* = 0.006) more frequently started adjuvant chemotherapy >4 weeks after surgery.

**Table 2:** Multivariate analysis of factors associated with TTC >4 weeks.

Characteristics	OR (95% CI)	*P*
**Age at diagnosis, years**		0.005
≥50 *vs* <50	1.32 (1.09–1.60)	
**Comorbidity**		<0.001
1 *vs* 0	1.38 (1.10–1.71)	0.004
≥2 *vs* 0	1.81 (1.32–2.47)	<0.001
**Breast surgery**		0.001
BCS *vs* Mastectomy*	1.14 (1.51–1.72)	
Histologic type		0.012
Non-IDC *vs* IDC	1.47 (1.09–1.98)	
**LVI**		0.060
Positive *vs* Negative	0.70 (0.49–1.02)	
**Molecular subtype**		<0.001
Luminal B/HER2- *vs* Luminal A	1.32 (0.99–1.77)	0.063
Luminal B/HER2 + *vs* Luminal A	1.74 (1.23–2.47)	0.002
TNBC *vs* Luminal A	0.74 (0.53–1.05)	0.087
HER2 + *vs* Luminal A	1.61 (1.15–2.27)	0.006

Abbreviations: TTC, time to adjuvant chemotherapy; OR, odds ratio; CI, confidence interval; BCS, breast-conserving surgery; IDC, invasive ductal carcinoma; LVI, lymphovascular invasion; HER2, human epidermal growth factor receptor 2; TNBC, triple negative breast cancer.

*57 (2.2%) out of 1989 patients received mastectomy + immediate breast reconstruction.

### Clinical outcomes in the whole population

After a median follow-up of 61.8 months, 454 (17.4%) patients experienced RFS events, including 53 (2.0%) local recurrences, 180 (6.9%) distant metastases, 44 (1.7%) second non-breast malignancies, 33 (1.3%) contralateral breast cancers, and 144 (5.5%) deaths.

**Figure 2:**
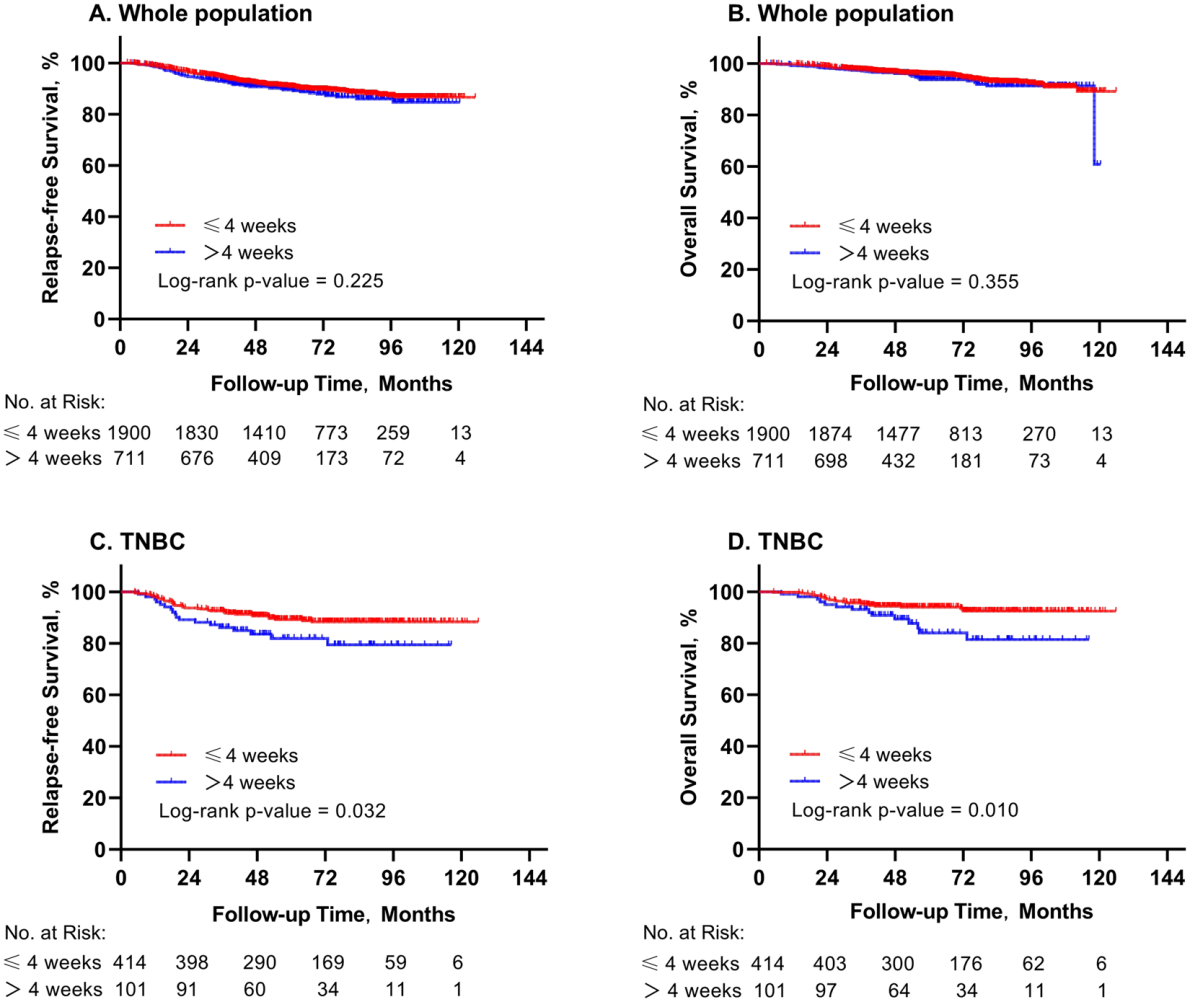
RFS and OS according to TTC in the whole population and in TNBC patients. Kaplan-Meier curves of RFS and OS according to TTC groups in the whole population (**A, B**) and in TNBC patients (**C, D**). Abbreviations: RFS, relapse-free survival; OS, overall survival; TTC, time to adjuvant chemotherapy; TNBC, triple negative breast cancer; HER2, human epidermal growth factor receptor 2; No., number.

Table 3 summarized the influencing factors associated with 5-year RFS and 5-year OS in the whole population. The 5-year RFS and OS were 89.0% and 94.0%. For patients with TTC ≤4 weeks and >4 weeks, the estimated 5-year RFS was 90.7% and 89.5%, respectively. No significant differences in RFS was observed between two TTC groups in the whole population (*P* = 0.225; Fig. 2A). When stratified by stage, TTC was not related to RFS either in stage I, II or III diseases (*P* = 0.957, 0.071, 0.479, Supplementary Figure S2A, S2C, S2E). Univariate and multivariate analyses demonstrated that, grade III tumor (HR, 1.56, 95% CI, 1.19 to 2.05, *P* = 0.001; Supplementary Table S3), greater tumor size (T2 *vs* T1: HR, 1.82, 95% CI, 1.41 to 2.35, *P* < 0.001; T3–4 *vs* T1: HR, 3.23, 95% CI, 1.81 to 5.77, *P* < 0.001), node involvement (HR, 2.19, 95% CI, 1.70 to 2.83, *P* < 0.001) and TNBC subtype (HR, 1.78, 95% CI, 1.08 to 2.93, *P* = 0.024) were significantly associated with impaired RFS.

**Table 3:** TTC, patient characteristics and survival outcomes.

Characteristics	5-year RFS	*P*	5-year OS	*P*
All patients	89.0%		94.0%	
**Age at diagnosis, years**		0.270		0.038
<50	91.7%		96.4%	
≥50	89.5%		94.4%	
**Comorbidity**		0.024		0.086
0	91.3%		96.0%	
1	88.2%		93.6%	
≥2	88.4%		92.9%	
**Breast surgery**		0.336		0.002
Mastectomy	90.2%		94.6%	
BCS	90.9%		97.3%	
**Histologic type**		0.662		0.700
IDC	90.4%		95.4%	
Non-IDC	90.2%		93.9%	
**Histologic grade**		<0.001		<0.001
I-II	92.8%		97.2%	
III	87.6%		93.0%	
Unknown	90.0%		94.4%	
**Pathological tumor size**		<0.001		<0.001
T1	93.7%		97.0%	
T2	86.8%		93.5%	
T3–4	77.7%		84.6%	
**Pathological node status**		<0.001		<0.001
Negative	93.4%		97.4%	
Positive	86.3%		92.3%	
**LVI**		0.001		<0.001
Negative	90.8%		95.7%	
Positive	85.2%		89.0%	
**Molecular subtype**		0.010		0.021
Luminal A	95.6%		98.8%	
Luminal B/HER2-negative	89.9%		95.4%	
Luminal B/HER2-positive	93.2%		96.0%	
TNBC	87.8%		92.2%	
HER2-positive	88.0%		94.8%	
**TTC, weeks**		0.225		0.355
≤4	90.7%		95.6%	
>4	89.5%		93.7%	

Abbreviations: TTC, time to adjuvant chemotherapy; RFS, relapse-free survival; OS, overall survival; BCS, breast-conserving surgery; IDC, invasive ductal carcinoma; LVI, lymphovascular invasion; HER2, human epidermal growth factor receptor 2; TNBC, triple-negative breast cancer; HR, hormone receptor.

Similarly, for patients with TTC ≤4 weeks and >4 weeks, the estimated 5-year OS was 95.6% and 93.7%, respectively. No significant differences in OS was observed between two TTC groups (*P* = 0.355; Fig. 2B). When stratified by stage, TTC was not related to OS either in stage I, II or III diseases (*P* = 0.468, 0.071, 0.555, Supplementary Figure S2B, S2D, S2F). Higher grade (*P* = 0.006), larger tumor size (*P <*0.001), node involvement (*P* < 0.001), and TNBC subtype (*P* = 0.019) were also associated with worse OS in the whole population.

### TTC and RFS by different molecular subtypes

RFS by different molecular subtypes were compared between TTC groups. The 5-year estimated RFS was 89.3% and 81.9% for TNBC patients who started chemotherapy ≤4 weeks and >4 weeks after surgery (*P* = 0.032; Fig. 2C). In addition, HER2-positive patients receiving trastuzumab showed similar RFS between TTC groups (5-year RFS 90.0% and 93.0%, *P* = 0.645). No significant differences in RFS according to TTC were seen among patients with the other BC subtypes (Supplementary Figure S3A, S3C, S3E, S3G).

**Figure 3:**
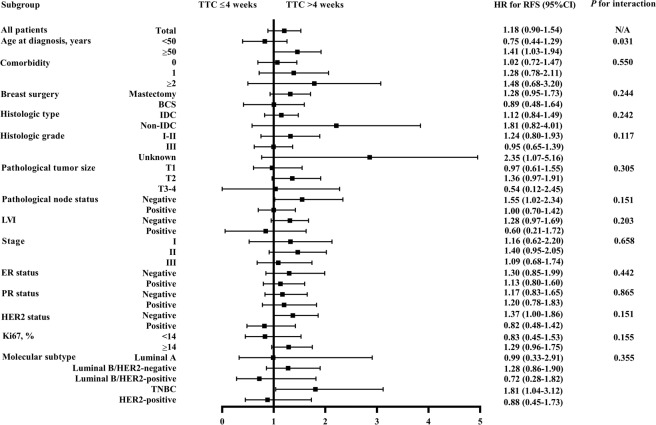
Interaction between TTC and patient clinicopathological characteristics on RFS. Abbreviations: TTC, time to adjuvant chemotherapy; HR, hazard ratio; RFS, relapse-free survival; CI, confidence interval; N/A, not available; BCS, breast-conserving surgery; IDC, invasive ductal carcinoma; LVI, lymphovascular invasion; ER, estrogen receptor; PR, progesterone receptor; HER2, human epidermal growth factor receptor 2; TNBC, triple-negative breast cancer; OS, overall survival.

Subgroup analysis found that age had a statistically significant interaction with TTC on RFS (*P* for interaction=0.031; Fig. 3). For patients aging 50 years or elder, TTC > 4 weeks was associated with worse RFS compared to TTC ≤4 weeks (HR, 1.41, 95%CI, 1.03 to 1.94, *P* = 0.035). Furthermore, for patients with TNBC, TTC> 4 weeks significantly increased recurrence risk compared to TTC ≤4 weeks (HR, 1.81, 95%CI, 1.04 to 3.12, *P* = 0.032), while such difference was not significant among other molecular subtypes (*P* for interaction=0.355).

Univariate and multivariate analyses (Supplementary Table S3) were then carried out to identify impact factors on RFS in patients with different molecular subtypes. For Luminal A patients, none of the clinico-pathological features, including TTC (*P* = 0.698) independently influence patient RFS. For Luminal B/HER2-negative patients, histologic grade (*P* < 0.001), tumor size (*P* = 0.002), and nodal status (*P* = 0.001), but not TTC (*P* = 0.339) were related to RFS. For Luminal B/HER2-positive patients, nodal status (*P* = 0.030), not TTC (*P* = 0.457) was associated with RFS. For HER2-amplified patients, age (*P* = 0.037), and nodal status (*P* = 0.005), but not TTC (*P* = 0.885) independently impact RFS.

Notably, for TNBC patients, univariate (Supplementary Table S4) and multivariate (Table 4) analyses demonstrated that, other than common risk factors including larger tumor size (*P* = 0.002), node involvement (*P <*0.001), and LVI (*P* = 0.046), longer TTC was independently related to higher risk of disease relapse in TNBC patients (HR, 1.89, 95% CI, 1.09 to 3.27, *P* = 0.024).

**Table 4:** Multivariate analysis of factors associated with RFS and OS in TNBC patients.

Characteristics	RFS		OS	
	HR (95% CI)	*P*	HR (95% CI)	*P*
**Histologic grade**		0.148		**0.041**
III *vs* I-II	1.84 (0.92–3.67)	0.084	2.36 (0.98–5.69)	0.056
Unknown *vs* I-II	1.11 (0.40–3.08)	0.845	0.62 (0.12–3.07)	0.554
**Pathological tumor size**		**0.002**		**0.022**
T2 *vs* T1	2.42 (1.38–4.24)	0.002	2.60 (1.09–6.21)	0.032
T3–4 *vs* T1	4.70 (1.37–16.08)	0.014	0.63 (0.13–3.12)	0.569
**Pathological node status**		<**0.001**		<**0.001**
Positive *vs* Negative	2.99 (1.77–5.07)		4.60 (2.35–9.04)	
**LVI**		**0.046**		**0.015**
Positive *vs* Negative	2.01 (1.01–3.98)		2.61 (1.20–5.69)	
**TTC, weeks**		**0.024**		**0.006**
>4 vs ≤4	1.89 (1.09–3.27)		2.49 (1.30–4.76)	

Abbreviations: RFS, relapse-free survival; OS, overall survival; TNBC, triple-negative breast cancer; HR, hazard ratio; CI, confidence interval; LVI, lymphovascular invasion; TTC, time from definitive surgery to initiation of adjuvant chemotherapy.

### TTC and OS by different molecular subtypes

For patients with TNBC, the 5-year estimated OS was 94.0% and 84.0% for patients who started chemotherapy ≤4 weeks and >4 weeks after surgery (*P* = 0.010; Fig. 2D). Meanwhile, HER2-positive patients receiving trastuzumab showed similar OS between TTC groups (5-year OS 95.3% and 98.9%, *P* = 0.065). No significant differences in OS according to TTC were seen among patients with the other BC subtypes (Supplementary Figure S3B, S3D, S3F, S3H).

**Figure 4:**
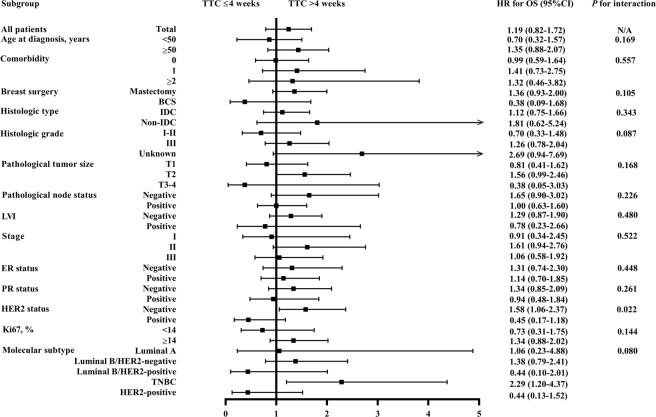
Interaction between TTC and patient clinicopathological characteristics on OS. Abbreviations: TTC, time to adjuvant chemotherapy; HR, hazard ratio; RFS, relapse-free survival; CI, confidence interval; N/A, not available; BCS, breast-conserving surgery; IDC, invasive ductal carcinoma; LVI, lymphovascular invasion; ER, estrogen receptor; PR, progesterone receptor; HER2, human epidermal growth factor receptor 2; TNBC, triple-negative breast cancer; OS, overall survival.

Subgroup analysis revealed that HER2 status had a statistically significant interaction with TTC on OS (*P* for interaction=0.022; Fig. 4). For HER2-negative patients, TTC > 4 weeks was associated with worse OS compared to TTC ≤4 weeks (HR, 1.58, 95% CI, 1.06 to 2.37, *P* = 0.026). In addition, for patients with TNBC, TTC > 4 weeks was significantly associated with worse OS compared to TTC ≤4 weeks (HR, 2.29, 95% CI, 1.20 to 4.37, *P* = 0.012), while such difference was not significant among other molecular subtypes (*P* for interaction=0.080).

Univariate and multivariate analyses (Supplementary Table S3) about impact factors on OS in patients with different molecular subtypes were also performed. For Luminal A patients, none of the clinico-pathological features, including TTC (*P* = 0.813) independently influence OS. For Luminal B/HER2-negative patients, histologic grade (*P* < 0.001), and nodal status (*P* = 0.003), but not TTC (*P* = 0.590) were related to OS. For Luminal B/HER2-positive patients, LVI (*P* = 0.034), not TTC (*P* = 0.289) was associated with OS. For HER2-amplified patients, nodal status (*P* = 0.004), but not TTC (*P* = 0.438) independently impact OS.

For TNBC patients, univariate (Supplementary Table S4) and multivariate (Table 4) analyses showed that, apart from higher grade (*P* = 0.022), node involvement (*P* < 0.001) and LVI (*P* = 0.015), prolonged TTC independently increased the risk of death in TNBC patients (HR, 2.49, 95% CI, 1.30 to 4.76, *P* = 0.006; Table 4).

## Discussion

In this current study, we included 2611 continuous BC patients and managed to demonstrate that age, comorbidities, breast surgery type, tumor pathologic type, and molecular subtype were independently associated with prolonged TTC. TTC wasn’t an independent impact factor for RFS or OS in the whole population, whereas TTC > 4 weeks was significantly associated with worse RFS (HR = 1.89) and OS (HR = 2.49) in TNBC patients. Our findings encouraged us to start adjuvant chemotherapy within 4 weeks after surgery in TNBC patients.

The optimal TTC remains uncertain for BC patients^8^. The median TTC of our study cohort was 20 (IQR 13–29) days, which was relatively shorter than other studies^9–12,14,15^. Till now, a 4-week interval from surgery to chemotherapy was most frequently adopted when assessing the impact of TTC^19^. Based on our clinical practice, we also chose 4 weeks as the cutoff value since it was the necessary duration to obtain a complete pathological report especially in those who needed additional FISH or multigene assay testing. Other TTC intervals evaluated in previous studies ranged from 2 weeks to 12 weeks^8–12^. For example, Gagliato *et al*. adopted TTC intervals of ≤30 days, 31–60 days, and ≥61 days^10^. Downing *et al*. divided TTC by 3 weeks, 3–6 weeks, 6–10 weeks, and >10 weeks^20^. Further explorations were warranted to decide the optimal TTC cutoff for BC patients.

Diverse impact factors have been identified for TTC, mainly consisted of patient factors, surgical/ tumor factors and socioeconomic factors. For example, it was reported that African American^21^, non-Hispanic black^12^, and Hispanic ethnicity^22^ were more likely to undergo longer treatment intervals compared with white patients. Elder age^23^, and more comorbidities^24^ also led to increased odds of longer TTC. In addition, surgical factors including postoperative complications and immediate autologous breast reconstruction significantly augmented the risk of prolonged TTC as well^25^. On the other hand, low socioeconomic status or underinsured patients were more likely to experience delayed treatment^12,22^. However, there was still limited data about the impact factors for prolonged TTC in the Chinese population. Overall, our findings were generally consistent with previous studies. As identified in our study, patient characteristics including elder age and more comorbidities, surgical factor like BCS procedure, as well as tumor factors like non-IDC histologic type, and HER2-positive subtype were significantly associated with chemotherapy initiation delay. One explanation would be that it would take longer time to assess the condition in elder patients with comorbidities before the initiation of chemotherapy. Meanwhile, for patients receiving BCS, they usually had relatively small tumor size and negative node, which needs more pathological-histological evaluation, including 21-gene RS testing, to make the final chemotherapy decision making. For example, 194/622 (31.19%) patients receiving BCS had a 21-gene RS test, which was higher than patients treated with mastectomy (255/1989, 12.82%). In addition, patients receiving EC-T regimen tended to start adjuvant chemotherapy sooner after surgery, since they were more likely to have node-positive, TN or HER2-positive diseases, and were often quicker to determine if chemotherapy was needed. Other chemotherapy regimens, like EC or TC, were more likely to be applied in node-negative, Luminal-like patients, and additional FISH or 21-gene RS testing were more often required before the initiation of chemotherapy. To note, some of the impact factors could be altered to improve the treatment efficacy, for example, to shorten the waiting duration of tumor histo-pathological evaluation including FISH test and 21-gene recurrence score testing. Moreover, for elderly patients, it would be better to collect a complete medical history with all comorbidities at the first time at consultation, to perform necessary clinical evaluations as soon as possible to rule out chemotherapy contraindications. Furthermore, TN patients with stage II-III disease are recommended to receive neoadjuvant therapy, which would shorten the TTC. Moreover, escalation therapy might be given if TNBC patients have residual disease after neoadjuvant therapy to further improve disease outcome.

The impact of TTC on patient survival has been widely evaluated with controversial results. Yu *et al*. demonstrated a reduced OS (HR, 1.15, 95% CI 1.03 to 1.28) and disease-free survival (HR, 1.16, 95% CI 1.01 to 1.33) with each 4‐week delay to the initiation of adjuvant chemotherapy^13^. Similarly, a meta-analysis which included 15410 patients reported that a 4-week increase in TTC was associated with a significant decrease in both OS (HR, 1.14; 95% CI, 1.10 to 1.17) and disease-free survival (HR, 1.14; 95% CI, 1.10 to 1.18)^26^. However, other individual studies found no survival impact with TTC up to 3 months^27–29^. Hershman *et al*. showed in a cohort of 5003 patients that those who started chemotherapy within 1, 2, or 3 months after surgery had similar survival^27^. Lohrisch C *et al*. conducted a retrospective analysis of 2594 patients and drew a similar conclusion^28^. In current study, we did not observe significant differences in terms of RFS or OS between two TTC groups in the whole population. One possible reason for such inconsistent findings was the different distribution of patient baseline characteristics between studies. For example, in the cohort of Yu *et al*., only 27.2% patients had T1 tumors, 52.4% patients had node-positive diseases, and 47.4% were Luminal A^13^. In comparison, 55.4% of our enrolled population had T1 tumors, 42.5% reported node involvement, and only 13.4% were Luminal A.

The influence of chemotherapy delay on survival was subtype-dependent^9,10^. For example, Colleoni *et al*. found that early TTC could improve the survival of premenopausal ER-negative BC patients rather than those with ER-positive tumors^30^. Notably, several recent studies linked late TTC to poor prognosis in TNBC patients^11,12,31^. Li *et al*. reported that a prolonged TTC >60 days significantly decreased the RFS in TNBC patients^11^. Another recent study showed that longer TTC was associated with worse OS in HR-negative patients, not in HR-positive ones^31^. In addition, TTC ≥91 days was shown to be associated with an increased risk in BC-specific death only in TNBC patients (HR, 1.53, 95% CI, 1.17–2.07), but not in patients with HR-positive or HER2-positive tumors^12^. Consistent with these findings, our data showed a substantially detrimental effect of adjuvant chemotherapy delay on the RFS (HR, 1.89, 95% CI, 1.09–3.27, *P* = 0.024) and OS (HR, 2.49, 95% CI, 1.30–4.76, *P* = 0.006) of TNBC patients. Since TNBC tumor had relatively aggressive biology, rapid proliferation, and relevant sensibility to chemotherapy^32,33^, TNBC patients were expected to derive most benefit from chemotherapy^34–36^. The results of our study strongly suggested an early initiation of adjuvant chemotherapy in TNBC patients. However, we didn’t find a substantial survival difference between TTC groups in patients with HER2-positive diseases, while it was previously reported that a TTC ≥61 days led to a worse survival (HR, 3.09; 95% CI, 1.49 to 6.39) compared to a TTC <30 days in HER2-positive subtype^10^. This could also be explained by the improved clinical outcomes with the application of combined anti-HER2 targeted therapy^37–39^. Moreover, delayed TTC was not associated with poor survival outcomes in patients with HR-positive tumors, which adhered to previous studies^9,10,12^. Potential explanations included efficient endocrine therapy and relatively superior survival in HR-positive patients^3,40–43^.

To our knowledge, this is the largest study in Chinese population to provide insights into the influence of TTC on the clinical outcomes of BC patients, and to evaluate the optimal treatment interval for TNBC subtype. Our highlights lied in the fact that we reported a large series of consecutive patients, and we performed subgroup analysis for all patients according to clinico-pathological characteristics and molecular type. We managed to reveal that TN patients had a poorer prognosis with a TTC of more than 4 weeks. Second, the database we applied, SJTU-BCDB, had a follow-up rate of 99.98% and an error rate of 0.01–0.02%, guaranteeing that our results were creditable and consistent with the real-world practice. Thirdly, all the patients enrolled had gone through multidisciplinary discussion and had received standardized adjuvant treatment, so as to avoid the bias caused by irregular treatment. As a result, our finding was important to shed light on a better treatment schedule for Chinese patients, especially for TNBC patients, who would better initiate chemotherapy within 4 weeks after surgery, to maximize treatment efficacy. Nevertheless, several limitations should be considered when interpreting our results. First, given the non-randomized and retrospective nature of the study design, the current study had potential bias and needed to be further validated in other cohorts and larger population. Second, the baseline characteristics of patients treated in the two study centers weren’t well balanced, since Jiaxing Center was a specific breast health center which treated younger patients with fewer comorbidities and earlier diseases, while Shanghai Center was a comprehensive center which treated elder patients with more comorbidities and more locally advanced diseases. We here presented a joint analysis of data because our two study centers had a long-term cooperation in clinical practice, had similar multidisciplinary management procedure and shared the same breast cancer database. As a result, the treatment that patients received was comparable between these two centers. Furthermore, no data regarding patient germline variant was currently available in this study and further study was warranted to give more information on genetic risk of the patients. Meanwhile, thanks to the application of systemic treatment, patients had relatively superior survival, and the recurrent events were relatively insufficient, which warrants a continuous long-term follow-up. On the other hand, further analysis should be conducted to identify the most optimal cutoff of TTC for TNBC patients as well as other molecular subtypes.

## Conclusions

In conclusion, prolonged TTC >4 weeks after BC surgery was not associated with worse survival outcomes in the whole BC patients. However, TTC >4 weeks may increase the risk of relapse or death in TNBC patients. Early initiation of adjuvant chemotherapy should be encouraged for TNBC patients, which deserves further clinical evaluation.

### Ethical approval

This study obtained approval from the independent Ethical Committees of Jiaxing University Affiliated Women  and Children Hospital, and Ruijin Hospital, Shanghai Jiao Tong University School of Medicine. All procedures involving human participants were in accordance with the ethical standards of the institutional and/or national research committee and with the 1964 Helsinki declaration and its later amendments or comparable ethical standards. Informed consent was acquired in each patient.

## Supplementary information

Supplementary information.

## Data Availability

The datasets analyzed during the current study are available from the corresponding authors on reasonable request.
