# Supplementary material for: Prolonged Time to Adjuvant Chemotherapy Initiation Was Associated with Worse Disease Outcome in Triple Negative Breast Cancer Patients
Source: Sci Rep. 2020 Apr 27;10:7029. doi: 10.1038/s41598-020-64005-4 (PMC7184599; doi:10.1038/s41598-020-64005-4)
Supplement: Supplementary file 1 — Supplementary information. [file 41598_2020_64005_MOESM1_ESM.pdf]

Prolonged Time to Adjuvant Chemotherapy Initiation Was Associated with Worse Disease Outcome in Triple Negative Breast Cancer Patients

Lifen Cai[#], Yiwei Tong[#], Xiaoping Zhu, Kunwei Shen, Juanying Zhu[*], Xiaosong Chen[*]

Supplementary Tables

Supplementary Table S1. Patient baseline characteristics by study center

Characteristics	All Patients (n=2611) No. (%)	Study Center: Jiaxing (n=519) No. (%)	Study Center: Shanghai (n=2092) No. (%)	*P*
Age at diagnosis, years				
Median age (IQR)	52 (45-60)	48 (44-56)	53 (46-60)	
<50	1065 (40.8)	290 (55.9)	775 (37.0)	**<0.001**
≥50	1546 (59.2)	229 (44.1)	1317 (63.0)	
Comorbidity [†]				**<0.001**
0	1869 (71.6)	430 (82.9)	1439 (68.8)	
1	537 (20.6)	74 (14.3)	463 (22.1)	
≥2	205 (7.9)	15 (2.9)	190 (9.1)	
Breast surgery				**<0.001**
Mastectomy	1989 (76.2)	470 (90.6)	1519 (72.6)	
BCS	622 (23.8)	49 (9.4)	573 (27.4)	
Histologic type				0.763
IDC	2386 (91.4)	476 (91.7)	1910 (91.3)	
Non-IDC	225 (8.6)	43 (8.3)	182 (8.7)	
Histologic grade				**<0.001**
I-II	1275 (48.8)	378 (72.8)	897 (42.9)	
III	1118 (42.8)	93 (17.9)	1025 (49.0)	

Unknown	218 (8.3)	48 (9.2)	170 (8.1)	
Pathological tumor size				**<0.001**
T1	1446 (55.4)	354 (68.2)	1092 (52.2)	
T2	1106 (42.4)	154 (29.7)	952 (45.5)	
T3-4	59 (2.3)	11 (2.1)	48 (2.3)	
Pathological node status				**<0.001**
Negative	1502 (57.5)	359 (69.2)	1143 (54.6)	
Positive	1109 (42.5)	160 (30.8)	949 (45.4)	
LVI				**<0.001**
Negative	2408 (92.2)	445 (85.7)	1963 (93.8)	
Positive	203 (7.8)	74 (14.3)	129 (6.2)	
ER status				**<0.001**
Negative	879 (33.7)	133 (25.6)	746 (35.7)	
Positive	1732 (66.3)	386 (74.4)	1346 (64.3)	
PR status				**<0.001**
Negative	1250 (47.9)	172 (33.1)	1078 (51.5)	
Positive	1361 (52.1)	347 (66.9)	1014 (48.5)	
HER2 status				**<0.001**
Negative	1932 (74.0)	441 (85.0)	1491 (71.3)	
Positive	679 (26.0)	78 (15.0)	601 (28.7)	
Ki67, %				**<0.001**
<14	763 (29.2)	244 (47.0)	518 (24.8)	
≥14	1848 (70.8)	274 (52.8)	1574 (75.2)	
Molecular subtype				**<0.001**
Luminal A	349 (13.4)	149 (28.7)	200 (9.6)	
Luminal B/HER2-negative	1068 (40.9)	202 (38.9)	866 (41.4)	
Luminal B/HER2-positive	317 (12.1)	36 (6.9)	281 (13.4)	
TNBC	515 (19.7)	90 (17.3)	425 (20.3)	

HER2-positive	362 (13.9)	42 (8.1)	320 (15.3)	
TTC				**<0.001**
≤4	1900 (72.77)	519 (100.0)	1381 (66.1)	
>4	711 (27.23)	0 (0.0)	711 (33.9)	

Abbreviations: IQR, interquartile range; BCS, breast-conserving surgery; IDC, invasive ductal carcinoma; LVI, lymphovascular invasion; ER, estrogen receptor; PR, progesterone receptor; HER2, human epidermal growth factor receptor 2; TNBC, triple negative breast cancer; TTC, time to adjuvant chemotherapy.

† Comorbidity score was calculated according to Charlson Comorbidity Index.

Supplementary Table S2. Adjuvant treatment information by TTC

Treatment	All Patients (n=2611)	TTC (weeks)		*P*
		≤4 (n=1900)	>4 (n=711)	
	No. (%)	No. (%)	No. (%)	
Chemotherapy regimen				
EC	277 (10.6)	217 (11.4)	60 (8.4)	<0.001
TC	710 (27.2)	437 (23.0)	273 (38.4)	
EC-T	1593 (61.0)	1228 (64.6)	365 (51.3)	
Others	31 (1.1)	18 (0.9)	13 (1.8)	
Radiation therapy				0.353
No	1269 (48.6)	934 (49.2)	335 (47.1)	
Yes	1342 (51.4)	966 (50.8)	376 (52.9)	
Targeted therapy				<0.001
No	2137 (81.8)	1613 (84.9)	524 (73.7)	
Yes	474 (18.2)	287 (15.1)	187 (26.3)	
Endocrine therapy				0.147
No	947 (36.3)	705 (37.1)	242 (34.0)	
Yes	1664 (63.7)	1195 (62.9)	469 (66.0)	

Abbreviations: TTC, time to chemotherapy; EC, epirubicin and cyclophosphamide; TC, taxane and cyclophosphamide; T, taxane.

Supplementary Table S3. Multivariate analysis of factors associated with RFS and OS in the whole population and by molecular subtype

Characteristics	RFS		OS	
	HR (95% CI)	*P*	HR (95% CI)	*P*
Whole population				
Age at diagnosis, years		0.882		0.229
≥50 *vs* <50	0.98 (0.75-1.28)		1.26 (0.87-1.82)	
Comorbidity		0.125		0.502
1 *vs* 0	1.31 (0.97-1.75)	0.075	1.22 (0.82-1.81)	0.331
≥2 *vs* 0	1.36 (0.89-2.08)	0.160	1.30 (0.73-2.32)	0.379
Histologic grade		**0.004**		**0.006**
III *vs* I-II	1.56 (1.19-2.05)	0.001	1.83 (1.26-2.66)	0.001
Unknown *vs* I-II	1.52 (0.97-2.38)	0.065	1.52 (0.83-2.78)	0.173
Pathological tumor size		**<0.001**		**<0.001**
T2 *vs* T1	1.82 (1.41-2.35)	<0.001	1.68 (1.18-2.39)	0.004
T3-4 *vs* T1	3.23 (1.81-5.77)	<0.001	3.89 (1.92-7.91)	<0.001
Pathological node status		**<0.001**		**<0.001**
Positive *vs* Negative	2.19 (1.70-2.83)		2.93 (2.05-4.20)	
LVI		0.087		0.089
Positive *vs* Negative	1.41 (0.95-2.07)		1.53 (0.94-2.51)	
Molecular subtype		**0.026**		**0.019**

LumB/HER2- *vs* LumA	1.57 (1.00-2.47)	0.051	1.50 (0.82-2.76)	0.193
LumB/HER2+ *vs* LumA	0.92 (0.51-1.65)	0.768	0.87 (0.39-1.93)	0.732
TNBC *vs* LumA	1.78 (1.08-2.93)	0.024	2.18 (1.13-4.20)	0.019
HER2+ *vs* LumA	1.51 (0.89-2.56)	0.129	1.18 (0.56-2.48)	0.668
Luminal A				
Age at diagnosis, years		0.389		0.780
≥50 *vs* <50	0.66 (0.25-1.71)		1.19 (0.35-4.13)	
Comorbidity		0.259		0.419
1 *vs* 0	1.56 (0.53-4.60)	0.423	1.63 (0.39-6.79)	0.504
≥2 *vs* 0	3.76 (0.74-19.08)	0.110	4.68 (0.44-50.27)	0.202
Histologic grade		0.554		0.964
III *vs* I-II	1.79 (0.63-5.11)	0.277	1.24 (0.26-5.93)	0.788
Unknown *vs* I-II	0.00 (0.00-)	0.973	0.00 (0.00-)	0.986
Pathological tumor size		0.207		0.182
T2 *vs* T1	1.72 (0.74-4.00)	0.207	2.27 (0.70-7.343)	0.172
T3-4 *vs* T1	5.42 (0.59-49.65)	0.135	9.36 (0.62-141.87)	0.107
Pathological node status		0.862		0.222
Positive *vs* Negative	1.08 (0.44-2.64)		0.44 (0.12-1.64)	
LVI		0.798		0.591
Positive *vs* Negative	1.23 (0.26-5.77)		1.67 (0.26-10.94)	
TTC		0.698		0.813

>4 *vs* ≤4	0.80 (0.26-2.46)		0.82 (0.16-4.23)	
Luminal B/HER2-negative				
Age at diagnosis, years		0.694		0.061
≥50 *vs* <50	1.09 (0.72-1.64)		1.83 (0.97-3.43)	
Comorbidity		0.472		0.573
1 *vs* 0	1.16 (0.74-1.83)	0.514	1.36 (0.75-2.47)	0.310
≥2 *vs* 0	1.44 (0.78-2.65)	0.242	1.28 (0.53-3.11)	0.586
Histologic grade		**<0.001**		**<0.001**
III *vs* I-II	1.97 (1.31-2.95)	0.001	2.40 (1.33-4.33)	0.004
Unknown *vs* I-II	3.08 (1.71-5.53)	<0.001	4.29 (3.06-8.96)	<0.001
Pathological tumor size		**0.002**		0.111
T2 *vs* T1	1.59 (1.07-2.34)	0.020	1.07 (0.63-1.84)	0.794
T3-4 *vs* T1	3.60 (1.61-8.08)	0.002	3.12 (1.07-9.12)	0.038
Pathological node status		**0.001**		**0.003**
Positive *vs* Negative	1.90 (1.29-2.80)		2.34 (1.35-4.06)	
LVI		0.876		0.478
Positive *vs* Negative	1.05 (0.55-2.04)		0.65 (0.20-2.12)	
TTC		0.339		0.590
>4 *vs* ≤4	1.22 (0.81-1.82)		1.17 (0.66-2.06)	
Luminal B/HER2-postive				
Age at diagnosis, years		0.999		0.886

≥50 *vs* <50	1.00 (0.38-2.72)		0.90 (0.23-3.59)	
Comorbidity		0.087		0.235
1 *vs* 0	2.30 (0.88-6.01)	0.088	3.10 (0.81-11.95)	0.100
≥2 *vs* 0	3.70 (0.94-14.56)	0.062	2.88 (0.30-27.82)	0.362
Histologic grade		0.981		0.773
III *vs* I-II	0.99 (0.38-2.54)	0.976	0.82 (0.22-3.06)	0.770
Unknown *vs* I-II	1.15 (0.24-5.53)	0.862	1.87 (0.19-18.52)	0.592
Pathological tumor size		0.319		0.203
T2 *vs* T1	2.11 (0.80-5.55)	0.131	4.32 (0.87-21.59)	0.074
T3-4 *vs* T1	0.00 (0.00-)	0.981	0.00 (0.00-)	0.991
Pathological node status		**0.030**		0.170
Positive *vs* Negative	2.84 (1.10-7.32)		2.68 (0.66-10.92)	
LVI		0.202		**0.034**
Positive *vs* Negative	2.14 (0.67-6.89)		5.02 (1.13-22.31)	
TTC		0.457		0.289
>4 *vs* ≤4	0.69 (0.26-1.83)		0.43 (0.09-2.05)	
HER2-amplified				
Age at diagnosis, years		**0.037**		0.292
≥50 *vs* <50	0.49 (0.25-0.96)		0.57 (0.20-1.62)	
Comorbidity		0.367		0.153
1 *vs* 0	0.73 (0.31-1.71)	0.469	0.22 (0.03-1.75)	0.153

≥2 *vs* 0	1.71 (0.63-4.69)	0.295	2.17 (0.55-8.46)	0.267
Histologic grade		0.523		0.778
III *vs* I-II	0.68 (0.35-1.33)	0.257	0.69 (0.25-1.93)	0.478
Unknown *vs* I-II	0.74 (0.17-3.35)	0.699	0.00 (0.00-)	0.982
Pathological tumor size		0.454		0.191
T2 *vs* T1	1.30 (0.66-2.55)	0.449	1.56 (0.52-4.74)	0.431
T3-4 *vs* T1	2.45 (0.54-11.19)	0.248	4.85 (0.88-26.63)	0.069
Pathological node status		**0.005**		**0.004**
Positive *vs* Negative	2.65 (1.35-5.21)		6.74 (1.84-24.66)	
LVI		0.958		0.683
Positive *vs* Negative	0.97 (0.33-2.88)		1.31 (0.03-1.75)	
TTC		0.885		0.438
>4 *vs* ≤4	1.05 (0.52-2.12)		0.60 (0.16-2.20)	

Abbreviations: RFS, relapse-free survival; OS, overall survival; HR, hazard ratio; CI, confidence interval; BCS, breast-conserving surgery; LVI, lymphovascular invasion; LumB, Luminal B; HER2, human epidermal growth factor receptor 2; LumA, Luminal A; TNBC, triple negative breast cancer; TTC, time to chemotherapy.

Supplementary Table S4. Univariate analysis of factors influencing RFS and OS in TNBC patients

Characteristics	RFS		OS	
	HR (95% CI)	*P*	HR (95% CI)	*P*
Age at diagnosis, years		0.721		0.918
≥50 *vs* <50	1.10 (0.65-1.86)		0.97 (0.51-1.83)	
Comorbidity		0.144		0.815
1 *vs* 0	1.54 (0.90-2.65)	0.115	1.21 (0.60-2.43)	0.603
≥2 *vs* 0	0.58 (0.18-1.86)	0.357	0.85 (0.26-2.79)	0.782
Breast surgery		0.214		0.181
BCS *vs* Mastectomy	0.69 (0.38-1.24)		0.59 (0.27-1.28)	
Histologic type		0.835		0.426
Non-IDC IDC	0.92 (0.44-1.94)		0.66 (0.23-1.85)	
Histologic grade		0.068		0.030
III *vs* I-II	2.11 (1.07-4.18)	0.032	2.55 (1.07-6.09)	0.035
Unknown *vs* I-II	1.25 (0.46-3.45)	0.662	0.69 (0.14-3.93)	0.643
Pathological tumor size		0.001		0.022
T2 *vs* T1	2.76 (1.59-4.81)	<0.001	2.39 (1.23-4.65)	0.010
T3-4 *vs* T1	4.49 (1.32-15.24)	0.016	3.74 (0.84-16.58)	0.082
Pathological nodal status		<0.001		<0.001
Positive *vs* Negative	3.52 (2.13-5.82)		5.16 (2.70-9.83)	

LVI		<0.001		<0.001
Positive *vs* Negative	3.41 (1.78-6.55)		4.38 (2.09-9.19)	
TTC, weeks		0.035		0.012
>4 *vs* ≤4	1.81 (1.04-3.12)		2.29 (1.20-4.37)	

Abbreviations: RFS, relapse-free survival; OS, overall survival; TNBC, triple-negative breast cancer; HR, hazard ratio; CI, confidence interval; BCS, breast-conserving surgery; IDC, invasive ductal carcinoma; LVI, lymphovascular invasion; TTC, time from definitive surgery to initiation of adjuvant chemotherapy.

Supplementary Figures

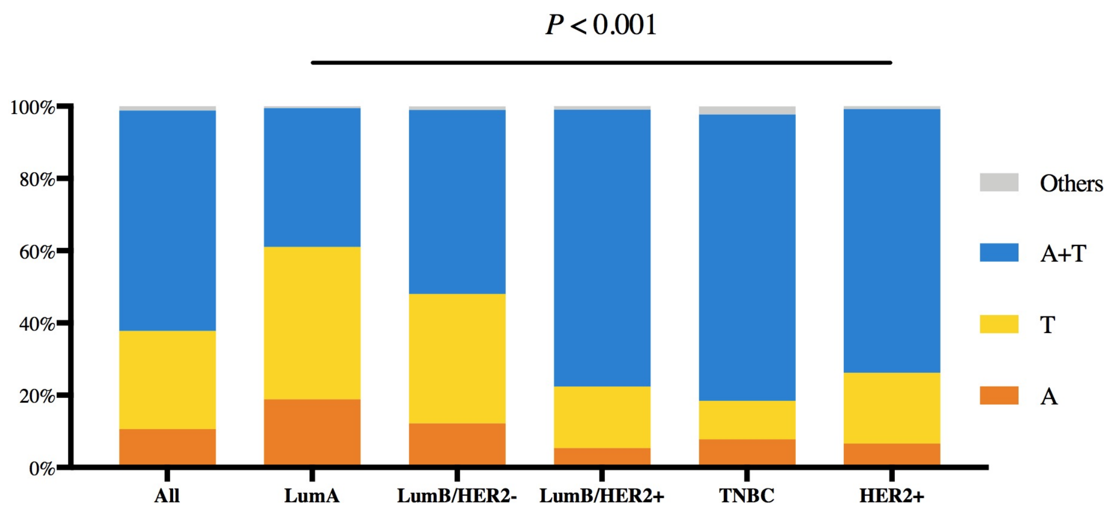

Supplementary Figure S1. Chemotherapy regimen by molecular subtypes.

Chemotherapy regimen in the whole population, in patients with Luminal A, Luminal B/HER2-negative, Luminal B/HER2-positive, TNBC and HER2-positive tumors.

Abbreviations: HER2, human epidermal growth factor receptor 2; TNBC, triple negative breast cancer; A, anthracyclins; T, taxanes; A+T, anthracyclins and taxanes.

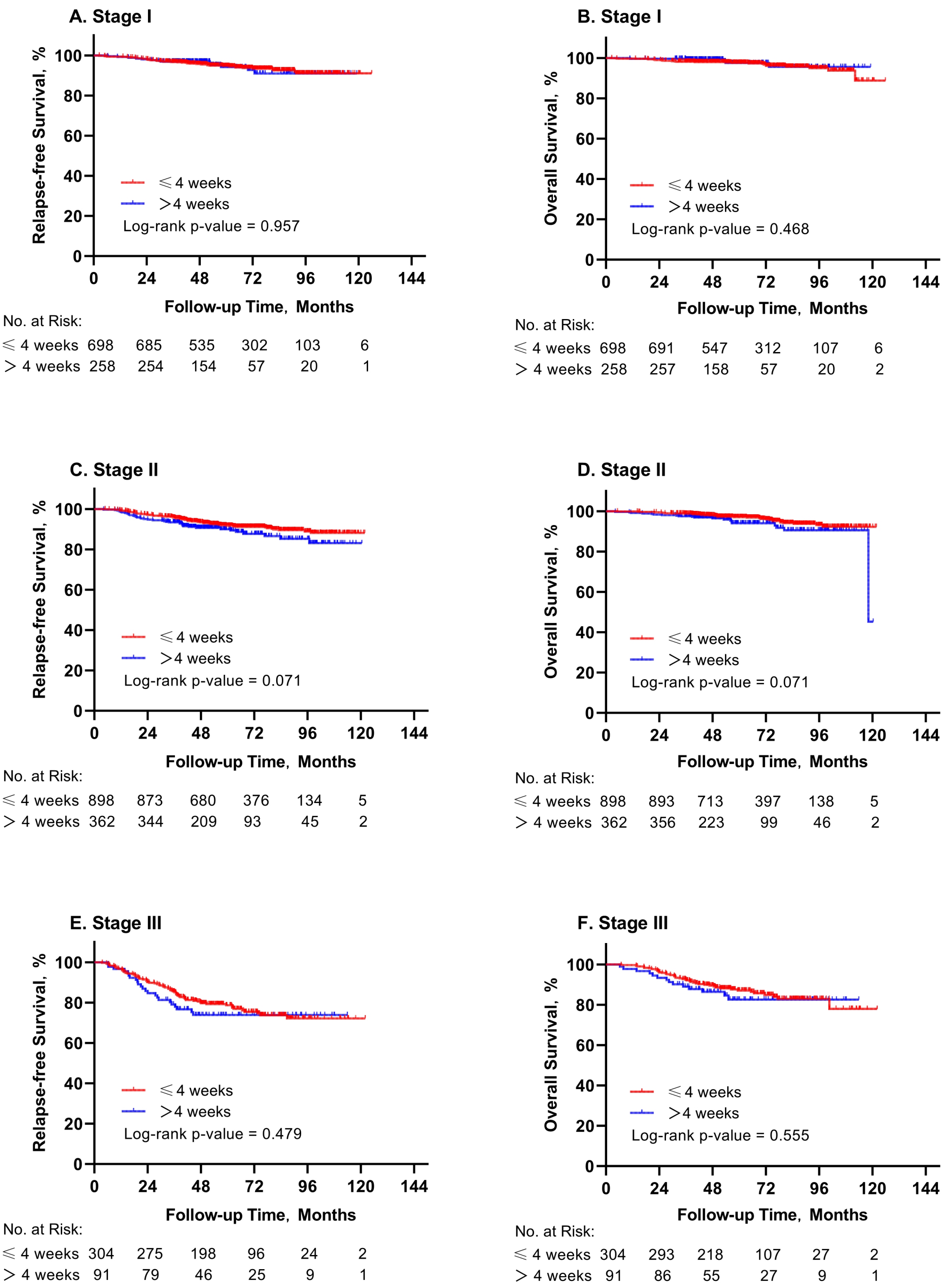

Supplementary Figure S2. RFS and OS according to TTC by disease stage.

Kaplan-Meier curves of RFS and OS according to TTC groups in patients with stage I (A, B), stage II (C, D), and stage III (E, F) diseases.

Abbreviations: RFS, relapse-free survival; OS, overall survival; TTC, time to adjuvant chemotherapy; HER2, human epidermal growth factor receptor 2; No., number.

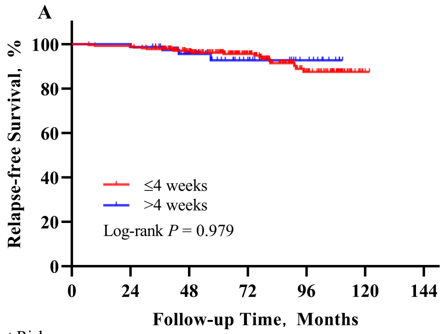

No. at Risk:

≤4 weeks	273	271	221	140	62	2
>4 weeks	76	76	48	23	11	1

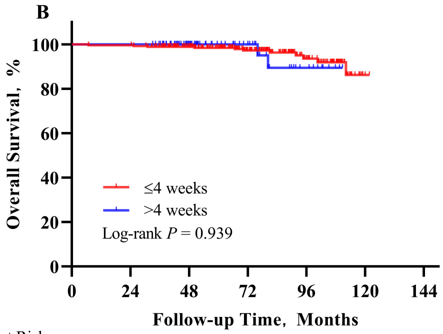

No. at Risk:

≤4 weeks	273	273	227	144	65	2
>4 weeks	76	76	50	26	11	1

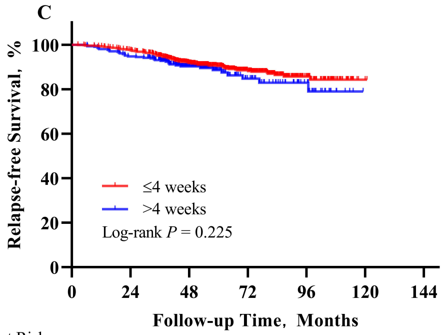

No. at Risk:

≤4 weeks	762	744	575	279	71	3
>4 weeks	306	291	161	55	25	1

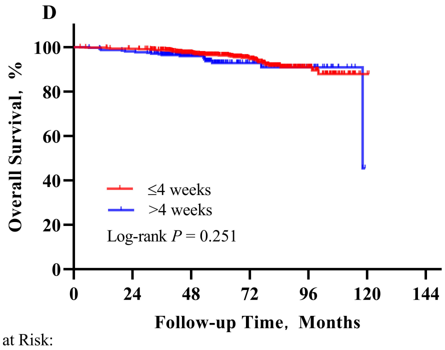

No. at Risk:

≤4 weeks	762	756	607	303	75	3
>4 weeks	306	301	171	58	25	1

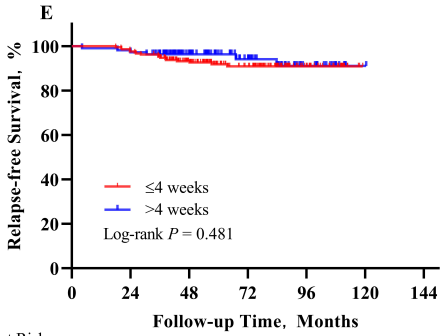

No. at Risk:

≤4 weeks	210	206	154	94	36	1
>4 weeks	107	105	68	40	18	2

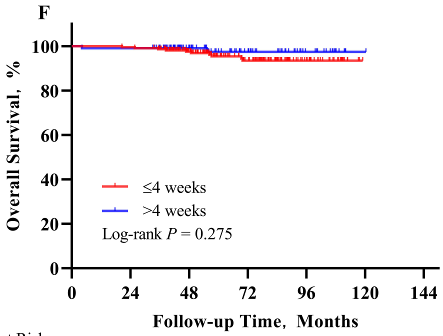

No. at Risk:

≤4 weeks	210	210	162	95	36	1
>4 weeks	107	107	70	41	19	2

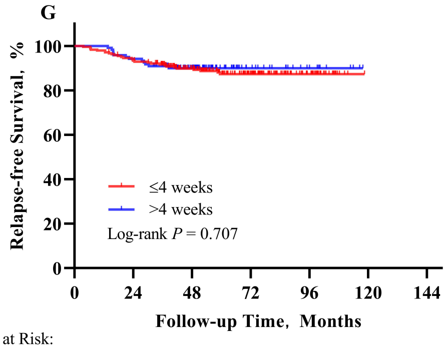

No. at Risk:

≤4 weeks	241	224	173	94	35	1
>4 weeks	121	115	74	24	10	1

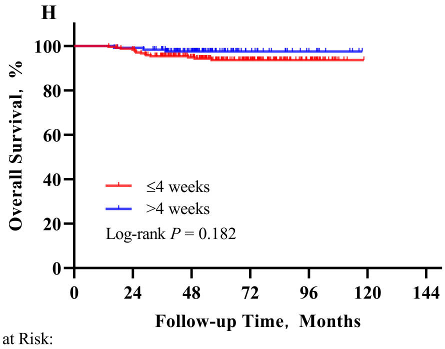

No. at Risk:

≤4 weeks	241	236	181	98	35	1
>4 weeks	121	120	78	25	10	1

Supplementary Figure S3. RFS and OS according to TTC by molecular subtypes.

Kaplan-Meier curves of RFS and OS according to TTC groups in patients with Luminal A (A, B), Luminal B/HER2-negative (C, D), Luminal B/HER2-positive (E, F) and HER2-positive (J, H) tumors.

Abbreviations: RFS, relapse-free survival; OS, overall survival; TTC, time to adjuvant chemotherapy; HER2, human epidermal growth factor receptor 2; No., number.
